# Mode-resolved reciprocal space mapping of electron-phonon interaction in the Weyl semimetal candidate *Td*-WTe_2_

**DOI:** 10.1038/s41467-020-16076-0

**Published:** 2020-05-26

**Authors:** Petra Hein, Stephan Jauernik, Hermann Erk, Lexian Yang, Yanpeng Qi, Yan Sun, Claudia Felser, Michael Bauer

**Affiliations:** 1 Institute of Experimental and Applied Physics, University of Kiel, Leibnizstraße 19, 24118 Kiel, Germany; 2 State Key Laboratory of Low Dimensional Quantum Physics, Department of Physics, Tsinghua University, Beijing, 100084 China; 3 Frontier Science Center for Quantum Information, Beijing, 100084 China; 4 School of Physical Science and Technology, ShanghaiTech University, Shanghai, 201210 China; 5 Max Planck Institute for Chemical Physics of Solids, Nöthnitzer Straße 40, 01187 Dresden, Germany

**Keywords:** Electronic properties and materials, Topological insulators

## Abstract

The excitation of coherent phonons provides unique capabilities to control fundamental properties of quantum materials on ultrafast time scales. Recently, it was predicted that a topologically protected Weyl semimetal phase in the transition metal dichalcogenide *Td*-WTe_2_ can be controlled and, ultimately, be destroyed upon the coherent excitation of an interlayer shear mode. By monitoring electronic structure changes with femtosecond resolution, we provide here direct experimental evidence that the shear mode acts on the electronic states near the phase-defining Weyl points. Furthermore, we observe a periodic reduction in the spin splitting of bands, a distinct electronic signature of the Weyl phase-stabilizing non-centrosymmetric *Td* ground state of WTe_2_. The comparison with higher-frequency coherent phonon modes finally proves the shear mode-selectivity of the observed changes in the electronic structure. Our real-time observations reveal direct experimental insights into electronic processes that are of vital importance for a coherent phonon-induced topological phase transition in *Td*-WTe_2_.

## Abstract (web summary)

It is predicted that topological phase transitions in quantum materials can be triggered by selective excitation of coherent phonons. Upon excitation of a shear mode, Hein et al. observe distinct perturbations of electronic Weyl semimetal fingerprints in *Td*-WTe_2_.

## Introduction

**Fig. 1:**
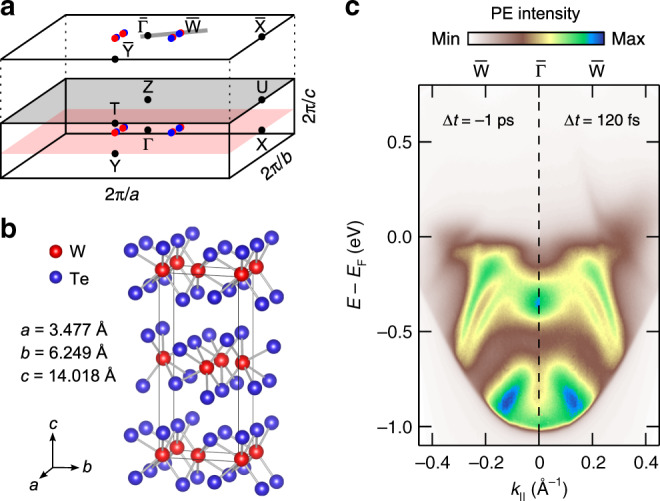
Crystal and electronic structure of *Td*-WTe_2_. **a** 3D Brillouin zone and its projection onto the (001) surface. Blue and red dots mark the approximate positions of the Weyl points as predicted in ref. ^8^. The gray line crossing $\overline{\Gamma }$ and $\overline{{\rm{W}}}$ indicates the momentum cut investigated in the present TRARPES study. **b** Orthorhombic unit cell with experimental lattice constants at *T* = 113 K^44^. **c** ARPES intensity maps in $\overline{\Gamma }$–$\overline{{\rm{W}}}$ direction before (Δ*t* = −1 ps) and after (Δ*t* = 120 fs) excitation with 827 nm pump pulses.

With their first experimental observation in 2015, topological Weyl semimetals (WSMs) attracted enormous attention^1–4^. In these materials, long-sought-after Weyl fermions are realized as quasi-particle excitations in condensed matter^5–7^. WSMs feature an unusual electronic structure with topologically protected crossing points in the bulk band structure, the so-called Weyl points. In the particular case of type-II WSMs, the Weyl points are located at touching points of electron and hole pockets close to the Fermi level *E*_F_^8–10^. The transition metal dichalcogenide *Td*-WTe_2_ was the first material proposed to support such a scenario and band structure calculations using low-temperature lattice parameters predicted the existence of four pairs of Weyl points in the Γ*XY* plane of the Brillouin zone (see Fig. 1a)^8^. Even though angle-resolved photoemission spectroscopy (ARPES) studies did not succeed in observing Weyl points in this material so far^11–17^, the observation of Weyl orbit related quantum oscillations and an anisotropic magnetoresistance^18^, the detection of Weyl points via scanning tunneling spectroscopy^19^, and the observation of an anisotropic Adler–Bell–Jackiw anomaly^20^ give strong experimental evidence that *Td*-WTe_2_ is indeed a type-II WSM.

The lack of an inversion center in the crystal structure is the prerequisite for a WSM phase in non-magnetic materials^8^. Recently, an ultrafast and reversible way of manipulating the structural symmetry of *Td*-WTe_2_ was demonstrated in a combined ultrafast electron diffraction (UED) and time-resolved second-harmonic generation (TRSHG) study^21^. Upon excitation with terahertz pump pulses, a coherent 0.24 THz interlayer shear excitation was observed that, at sufficiently high excitation densities, drives a structural phase transition from the non-centrosymmetric *Td* ground state of the material (see Fig. 1b) into a meta-stable centrosymmetric $1T^{\prime}$(*) phase^21^. Band structure calculations imply the periodic modulation of the Weyl point intra-pair separation upon excitation of the shear mode. Even more, the complete annihilation of the Weyl points is expected as the material undergoes the transition into the $1T^{\prime}$(*) phase. However, experimental data on how the shear mode affects the band structure of *Td*-WTe_2_ is still lacking.

Here, we present a time-resolved ARPES (TRARPES) study on the electronic structure response of *Td*-WTe_2_ to the excitation of coherent phonons. Upon absorption of 827 nm femtosecond laser pulses, we observe in the TRARPES data clear oscillations in photoemission (PE) intensity, band positions, and bandwidths. The comparison with Raman spectroscopy^22–24^ and optical pump-probe spectroscopy results^25,26^ allows assigning these oscillations to the excitation of five different A_1_ optical phonon modes, with one of them being the interlayer shear mode mentioned above. An energy- and momentum-resolved Fourier transformation of the TRARPES data enables us to perform a phonon mode-selective analysis of the electronic structure response. We observe that the excitation of the interlayer shear mode periodically modulates occupied bands that are spin split due to the broken inversion symmetry of the crystal including a hole pocket that is directly involved in the formation of the Weyl points at low temperatures^27^. Even more, we observe that also the PE signal from the energy-momentum region of the Weyl points shows clear oscillations at the shear mode frequency. Although the presented experiments were performed at room temperature, for which theory excludes the presence of Weyl points^11^, our results give direct experimental support for a coherent phonon mediated control of the electronic structure relevant for the Weyl physics in *Td*-WTe_2_.

## Results

### Coherent phonon excitation

**Fig. 2:**
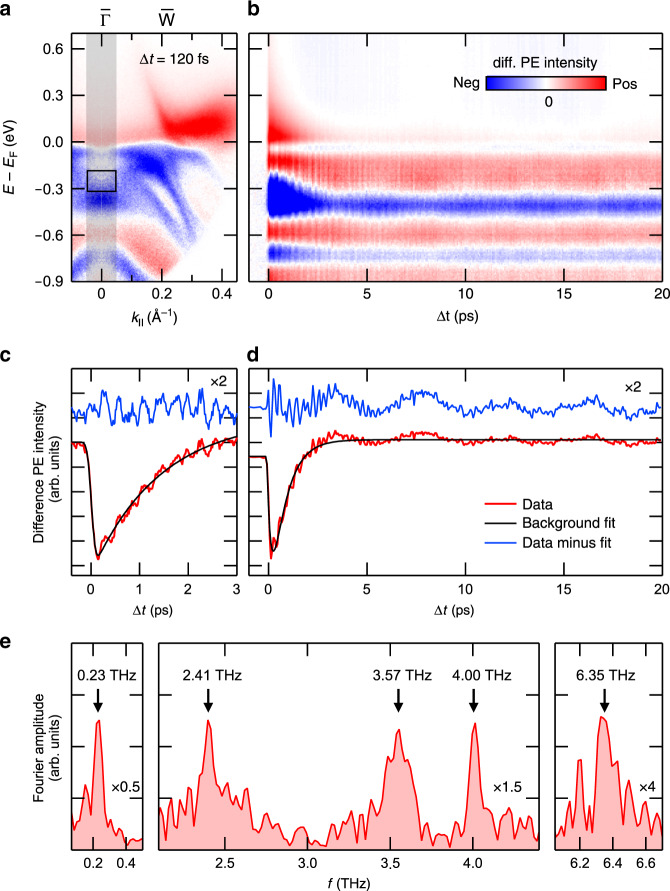
TRARPES results of *Td*-WTe_2_ along $\overline{\Gamma }$-$\overline{{\rm{W}}}$. **a** ARPES difference intensity map derived from the TRARPES data at Δ*t* = 120 fs and Δ*t* = −1 ps shown in Fig. 1c. **b** Transient difference EDCs derived from the ARPES difference intensity map by momentum integration of the gray-shaded area in **a**. **c**, **d** PE intensity transients of the integration region marked by the black box in **a** before (red) and after (blue) background subtraction (Supplementary Note 3). Blue curves are offset and scaled by a factor of 2 for clarity. **e** Fourier amplitude spectrum of the intensity transient shown in **d**. Note the different scaling of the data.

Figure 1c shows TRARPES data of *Td*-WTe_2_ recorded before the optical excitation (Δ*t* = −1 ps) in comparison with data recorded at a pump-probe delay of Δ*t* = 120 fs. Overall, the spectra are consistent with ARPES and TRARPES data recorded at a similar probe photon energy of *hν* ≈ 6 eV^11,15,28^. In agreement with previous TRARPES studies, we observe the transient population of an electron pocket above *E*_F_ in response to the near-infrared (NIR) excitation^28–30^. A difference intensity map of the two ARPES spectra (see Fig. 2a) emphasizes the presence of spectral changes also below *E*_F_. Part of the observed transient reduction of spectral weight (blue areas) results from the depopulation of the occupied bands due to the absorption process. However, the observation of an increase in spectral weight in some regions below *E*_F_ (red areas) hints also to the presence of transient band renormalization processes.

Figure 2b shows transient difference energy distribution curves (EDCs) around the $\overline{\Gamma }$ point as a function of Δ*t*. Above *E*_F_, the data clearly reveals the presence of an excited carrier population that decays on a timescale of several picoseconds. Furthermore, part of the transient spectral changes are periodically modulated indicative for the excitation of at least two coherent phonon modes exhibiting oscillation periods in the few hundred femtoseconds and few picoseconds range, respectively. Significant long-lived spectral changes survive the damping of the coherent phonon modes and show barely any changes even at delays of 400 ps (Supplementary Note 1). Our observations qualitatively resemble the findings of previous time-resolved studies of *Td*-WTe_2_: electronic excitation and relaxation processes were studied in detail using TRARPES^28–30^ and time-resolved reflectivity (TRR) measurements^25^. The excitation of coherent phonons was observed in TRR^25,26^ and TRSHG experiments^21^ as well as via UED^21^, but, notably, in none of the past TRARPES studies^28–30^. Also the observed long-lived spectral changes are compatible with the findings of different other studies^21,25^. In the following, we will exclusively focus on the analysis of the coherent phonon oscillations and their impact on the electronic structure of *Td*-WTe_2_.

Figure 2c, d shows PE intensity transients around $\overline{\Gamma }$ and *E* − *E*_F_ = −0.25 eV. The data were taken with different sampling rates (60 and 15 THz) and cover different delay ranges (3 and 20 ps) to separately illustrate both the high- and low-frequency contributions to the PE intensity modulations (Supplementary Note 2). Raw data (red lines) were fitted with an exponential model function (Supplementary Note 3) to account for the carrier population dynamics (black lines) and to extract the pure oscillatory part of the signals (blue lines). The beating of the 3 ps range data clearly reveals the presence of more than one high-frequency mode. Additionally, a well-separated low-frequency modulation can be identified in the 20 ps range data. Figure 2e shows a Fourier amplitude spectrum of the 20 ps range intensity transient. Overall, we find main peaks at 0.23, 2.41, 3.57, 4.00, and 6.35 THz indicative for the excitation of at least five different coherent phonon modes. A comparison with the results of Raman studies of WTe_2_ and MoTe_2_ allows assigning all frequencies to A_1_ optical phonon modes^22–24,31^ that belong to the group of *m*-modes with the atoms vibrating in the *bc*-mirror plane of the unit cell^31^. Of particular relevance is the assignment of the 0.23 THz mode, which is responsible for the distinct long-periodic spectral modulations visible in Fig. 2b, d: In agreement with UED, TRSHG, and TRR results^21,25,26^, and predictions from Raman studies^22–24,31^, we assign this frequency to the low-energy optical phonon interlayer shear mode along the *b* axis (see below). The excitation of this mode periodically drives WTe_2_ from its non-centrosymmetric *Td* structure towards a centrosymmetric $1T^{\prime}$(*) structure. It is therefore expected to periodically modulate the spin splitting of bands^27^ as well as the Weyl point intra-pair separation in this material^21^.

### Mode-resolved electronic structure response

**Fig. 3:**
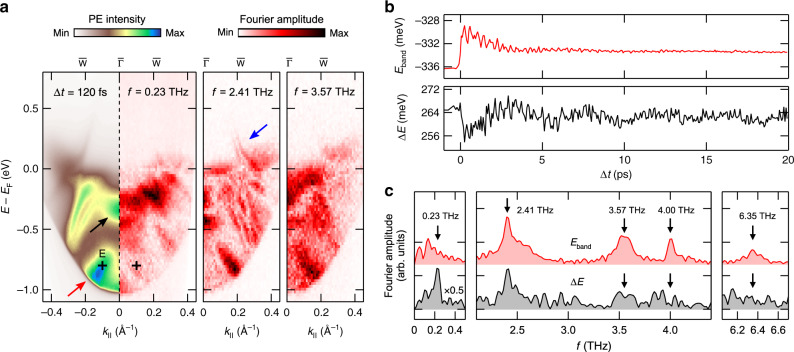
Phonon mode-selective Fourier amplitude analysis of the TRARPES data. **a** ARPES intensity map at Δ*t* = 120 fs in comparison to Fourier maps at different phonon frequencies. Fourier maps were generated from ARPES intensity maps that were binned over regions of 23 meV × 0.01 Å^−1^ in order to improve the signal statistics. Arrows indicate energy-momentum regions that are discussed in more detail in the main text. The cross marks the position of ROI E. Fourier maps of the 4.00 THz mode and the 6.35 THz mode are shown in Supplementary Fig. 5. **b** Transient peak energy *E*_band_ and peak width Δ*E* (FWHM) of the ARPES signal marked by the black arrow in **a**. The data result from Gaussian fits of the transient EDCs at $\overline{\Gamma }$. PE intensity was integrated over a momentum window of 0.05 Å^−1^. **c** Fourier amplitude spectra of the transients shown in **b**. The peaks below 0.23 THz cannot be assigned to any phonon modes in *Td*-WTe_2_. Their origin is discussed in Supplementary Note 2.

Figure 3a shows the results of an energy- and momentum-resolved Fourier analysis of the TRARPES data (Supplementary Note 4) illustrating the phonon mode-selectivity of the electronic structure response. The figure separately displays energy-momentum maps of the Fourier amplitudes of the 0.23, 2.41, and 3.57 THz coherent phonon excitations in comparison to the ARPES intensity map at Δ*t* = 120 fs. The data clearly illustrates the band-selectivity of the phonon excitations, as seen for instance for the band marked by the red arrow. A more detailed analysis shows, furthermore, that even in the case that the same band is affected by several modes, its response can be quite different. This is illustrated by band energy (*E*_band_) and bandwidth (Δ*E*) transients (Fig. 3b) and their Fourier amplitude spectra (Fig. 3c) for the feature in the TRARPES map centered at $\overline{\Gamma }$ at an energy *E* − *E*_F_ ≈ −0.3 eV marked by the black arrow. The excitation of the four high-frequency modes results in small but clearly detectable shifts in *E*_band_, which becomes particularly evident in the Fourier amplitude spectrum of the band energy transient. For the excitation of the 0.23 THz interlayer shear mode, such a band shift, if present at all, stays below the detection limit. In contrast, a finite and—also in comparison to the other modes—now significant contribution of the shear mode to the broadening of the band is evident from the Δ*E* transient as well as from the 0.23 THz peak in the corresponding Fourier amplitude spectrum. It is finally noteworthy that the Fourier analysis of the TRARPES data not only shows a finite signal amplitude below *E*_F_ but also at energies above *E*_F_ up to *E* − *E*_F_ ≈ 0.3 eV (see blue arrow). This sensitivity partly results from the laser excitation, which transiently heats up the electron gas so that states above *E*_F_ are substantially populated even on timescales of several tens of picoseconds.

In comparison to the ARPES intensity maps, the Fourier maps exhibit additional fine structures showing spectral details even below the 40 meV energy resolution of the experiment. The fine structure is particularly striking in the 2.41 THz Fourier map. A comparison with experimental data of a low temperature, high-resolution ARPES study^11^ shows that the fine structure resembles in large part the band structure of *Td*-WTe_2_. We account non-linearities in the dynamical signal response to the coherent phonon excitation being responsible for this resolution enhancement. More details are given in Supplementary Note 5.

Apart from the mode-selective amplitude analysis, we also performed a mode-selective phase analysis of the transient peak energies (Supplementary Note 6). The four high-frequency oscillations all show a cosinusoidal behavior with respect to time zero of the experiment implying a displacive excitation of the coherent phonons^32^. For the 0.23 THz mode, we observe, in contrast, a sinusoidal modulation of the peak energies, in agreement with UED and TRSHG results using THz and NIR excitation pulses, respectively^21^. Typically, such a response is associated with an impulsive Raman stimulated excitation mechanism^33^. It should be added that for the excitation of the interlayer shear mode in *Td*-WTe_2_ using THz pulses a field-driven photo-doping process was alternatively suggested to explain the observed sinusoidal response^21^.

### Shear mode excitation and electronic structure

In further discussion, we will focus on the excitation of the 0.23 THz interlayer shear mode periodically modulating the WTe_2_ crystalline *Td* structure towards a centrosymmetric $1T^{\prime}$(*) structure^21^. Based on values given in ref. ^21^ and under consideration of a TRR study at 800 nm excitation^26^, we estimate the shear displacement amplitude at the excitation fluence applied in our experiment to be in the order of 1 pm (Supplementary Note 7).

**Fig. 4:**
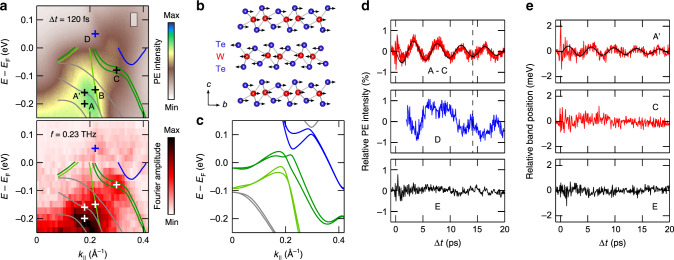
Electronic structure response to the shear mode excitation. **a** Close-ups of the ARPES intensity map near *E*_F_ at Δ*t* = 120 fs (top) and the corresponding 0.23 THz Fourier map (bottom). The data is overlaid with experimental band dispersions from ref. ^11^. Surface bands were omitted for the sake of clarity. Crosses mark positions of ROI A–D and A′. The Fourier map was generated from ARPES intensity maps that were binned over regions of 23 meV × 0.01 Å^−1^. **b** Schematic illustration of the atomic motion upon excitation of the interlayer shear mode^31^. **c** Calculated bulk band structure along the Γ–*X* direction. **d** PE intensity transients of ROI A–E. The transients of ROI A–C (top graph) can barely be discerned and are all displayed in red. The size of the integration area used to generate the transients is indicated by the gray-shaded box in the ARPES data shown in **a**. **e** Peak shift analysis of ROI A′, C, and reference region E marked in Fig. 3a. All transients in **d** and **e** are background-corrected (Supplementary Note 3). The black lines in the top graphs of **d** and **e** are fits of a damped sinusoidal function.

Figure 4a shows close-ups of the ARPES intensity map at Δ*t* = 120 fs and the 0.23 THz Fourier map of Fig. 3a. The data is overlaid with the bulk band structure of *Td*-WTe_2_ along the $\overline{\Gamma }$–$\overline{{\rm{X}}}$ direction experimentally determined in a high-resolution ARPES study at *hν* = 6.01 eV^11^. The color coding was chosen to discriminate between an electron pocket (blue), two hole pockets (green), and further bulk bands (gray). Calculated band structure data along Γ–*X* is shown for comparison in Fig. 4c. Due to thermal broadening and the limited energy resolution of our experiment in combination with strong variations in the spectral weight among the different bands^11^, it is difficult to discern the individual bands in the ARPES intensity map. However, even though broadened, the dispersing amplitude maxima in the Fourier map match part of the experimental band structure data strikingly well. The main amplitude maximum at *k*_∥_ ≈ 0.2 Å^−1^ shows a branching (see also Fig. 3a) that follows the dispersion of the lower hole pocket and the two gray-colored lower bulk bands, respectively. The data also reproduces the dispersion of the upper hole pocket up to energies close to *E*_F_ with a distinct amplitude maximum in the plateau region at *k*_∥_ ≈ 0.3 Å^−1^. Above *E*_F_, we observe two weak but distinct local amplitude maxima at *k*_∥_ ≈ 0.22 Å^−1^ and *k*_∥_ ≈ 0.34 Å^−1^ with the former one being approximately located at the surface projection of the Weyl points marked by the blue cross in Fig. 4a^8,19^.

Within the energy-momentum region probed in our experiments, different ARPES studies reported also on the observation of surface states^11–13,15^. Owing to the limited energy resolution and the fact that our experiments were performed at room temperature, no distinct signatures of these surface states could be observed in our ARPES intensity maps. A comparison with the experimental surface state data of ref. ^11^ showed, furthermore, that also the 0.23 THz Fourier map yields no clear indication for a response of the surface states to the excitation of the shear mode. In Fig. 4a, we therefore omitted the inclusion of the surface bands for the sake of clarity. A comparison including surface bands is, however, provided in Supplementary Note 8.

For further analysis, we selected four regions of interest (ROI) in the Fourier maps at positions indicated by the crosses in Fig. 4a. ROI A, B, and C are located at Fourier amplitude maxima. ROI B and C are furthermore intersected by the lower and upper hole pocket, respectively, whereas ROI A is positioned in between the two lowest bulk bands. ROI D is centered on the surface projection of the Weyl points above *E*_F_. For reference, we selected an additional ROI E (see Fig. 3a) at a local intensity maximum in the ARPES intensity maps that, however, shows a negligible amplitude in the 0.23 THz Fourier map. Where possible, we evaluated for the different ROI the transient evolution of the PE intensity, peak energy, and peak width in response to the excitation of the interlayer shear mode. For all ROI, we chose a signal integration area of 50 meV × 0.025 Å^−1^.

Results of the PE intensity analysis are summarized in Fig. 4d. For ROI A, B, and C, we observe clear periodic modulations at the frequency of the shear mode. Relative amplitude and phase of the PE intensity transients of the ROI match each other extremely well (see top graph of Fig. 4d) and can be described by a damped *π*-shifted sinusoidal function with a relative amplitude in the order of 1% (see full black line). Also the PE intensity of ROI D, which probes the Weyl point region, shows clear oscillations at 0.23 THz in spite of some signal distortions at Δ*t* ≈ 7.5 ps resulting from limitations of the signal background subtraction (Supplementary Note 3). Notably, the oscillatory response of ROI D shows a *π*-phase shift with respect to ROI A, B, and C as indicated by the black dashed line and also with respect to the second amplitude maximum above *E*_F_ at *k*_∥_ ≈ 0.34 Å^−1^ (Supplementary Note 9). Contrary to ROI A–D, the transient PE intensity signal of ROI E exhibits no evidence for a response to the shear mode as expected from the vanishing signal in the Fourier map.

A peak shift analysis is only possible for ROI that are located at signal maxima in the ARPES intensity maps. This only applies to ROI C and E. To account for potential spectral shifts affecting ROI A, we selected a nearby PE intensity maximum (ROI A′) separated by  ≈40 meV and intersected by the center bulk band. Distinct PE intensity maxima that could be associated with ROI B and D could not be identified. Results of the peak shift analysis are shown in Fig. 4e. A distinct shift in the peak energy at the shear mode frequency is only observed for ROI A′. The oscillation follows a damped sinusoidal function with an amplitude of approximately 1 meV. The data reveals no detectable peak shift at the shear mode frequency for ROI C and E. The analysis of the peak widths for ROI A′, C, and E did not yield any resolvable changes at the shear mode frequency. However, a detailed Fourier analysis of ROI C shows indirect evidence for subtle changes in the peak width as will be discussed below.

## Discussion

Reference ^27^ reports on the evolution of the band structure of WTe_2_ as the crystalline structure undergoes a complete transition from the *Td* phase to the centrosymmetric $1T^{\prime}$ phase upon external charge doping. The calculations show that the changes in the band structure predominantly result from changes in the interlayer interaction that is in an analog manner periodically modulated upon excitation of the 0.23 THz interlayer shear mode following the photoexcitation with ultrashort NIR pulses. More specifically, such a transition is expected to reduce and finally annihilate the spin splitting of the different bands near *E*_F_ as the inversion symmetry of the crystalline structure is recovered. These findings imply that the oscillatory part of the PE intensity transients from the two hole pockets (ROI B and C) results from a periodic modulation of the spin splitting due to the shear mode excitation. Notably, for the upper hole pocket the Fourier amplitude is indeed maximum in the region at which the experimental band structure data implies the largest spin splitting and also for the lower hole pocket, band structure calculations predict a considerable spin splitting (see Fig. 4c).

**Fig. 5:**
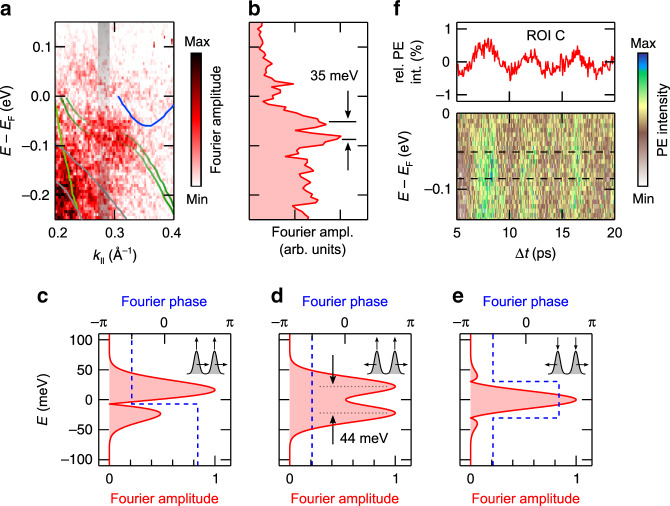
Fourier amplitude modulation near ROI C. **a** Close-up of the 0.23 THz Fourier map near ROI C. The data is overlaid with experimental band dispersions from ref. ^11^. For better visibility of the Fourier amplitude modulation near ROI C, the spin split upper hole bands are kept semi-transparent in this region. The energy-momentum cut displayed in **b** is indicated. The Fourier map was generated from ARPES intensity maps that were binned over regions of 6 meV × 0.0025 Å^−1^. **b** Fourier amplitude as a function of binding energy extracted from the Fourier map along the energy-momentum cut indicated in **a**. The separation of the amplitude maxima near ROI C is indicated. **c**–**e** Fourier amplitude and Fourier phase as a function of binding energy resulting from the simulations of **c** scenario (i), **d** scenario (ii), and **e** scenario (iii). The pictograms in the graphs schematically illustrate the considered scenarios. Details are given in the text and in Supplementary Note 10. The separation of the Fourier amplitude maxima for scenario (ii) is indicated. **f** Color-coded plot of experimental EDCs along the evaluated energy-momentum cut near ROI C as a function of Δ*t*. The dashed lines mark the binding energies of the two Fourier amplitude maxima indicated in **b**. The top graph displays for comparison the PE intensity transient of ROI C.

A more detailed inspection of the Fourier amplitude signal near ROI C further confirms that the shear mode excitation modulates the spin splitting. Figure 5a shows the 0.23 THz Fourier map near ROI C. In comparison to Figs. 3a and 4a, the original ARPES data were in this case binned over smaller energy-momentum regions for further data processing so that some more details become visible at the cost of signal statistics. The pronounced maximum in the Fourier map at ROI C appears now split by a weak but distinct amplitude minimum that follows the experimental band structure data from ref. ^11^ strikingly well. A constant momentum cut across the Fourier amplitude maximum (Fig. 5b) confirms this splitting and yields a separation of the resulting two Fourier amplitude maxima of  ≈35 meV. To gain insight into the origin of this Fourier amplitude modulation, we performed simulations mimicking the following three potential scenarios: (i) a rigid shift of the spin-split bands accompanying an overall PE intensity oscillation, (ii) a modulation of the spin splitting that is in phase with a PE intensity oscillation, i.e., a decrease (increase) of the spin splitting is accompanied by a decrease (increase) of the PE intensity, and (iii) a modulation of the spin splitting that is out of phase with a PE intensity oscillation. Details on the simulations, including the choice of parameters, are described in Supplementary Note 10.

Figure 5c–e shows the resulting Fourier amplitudes and phases as a function of energy for the three scenarios. Whereas the amplitude modulation calculated for scenario (iii) clearly deviates from the experimental results, a reasonable match is observed for scenario (i) and scenario (ii). However, inspection of the phase behavior shows that only scenario (ii) can consistently reproduce the experimental data: Fig. 5f depicts a color-coded plot of experimental EDCs along the evaluated energy-momentum cut near ROI C as a function of Δ*t*. The data reveal an in-phase response of the PE signal independent of binding energy across the two Fourier amplitude maxima (see dashed lines), in agreement with the constant phase expected for scenario (ii) and contrary to the *π*-phase shift right at the Fourier amplitude minimum expected for scenario (i). Notably, the simulation reproduces the experimental amplitude modulation as well as the separation of the two amplitude maxima also quantitatively strikingly well and implies that in our experiment the spin splitting is periodically modulated by the excitation of the shear mode at an amplitude in the order of 1 meV (see Supplemental Note 10). It is finally interesting to also consider the initial phase of the oscillation in the spin splitting. The excitation of the shear mode results in an initial reduction of the PE intensity in ROI C (see Fig. 4d). For the case of scenario (ii), this implies an initial reduction of the spin splitting, i.e., an initial shear motion towards the centrosymmetric $1T^{\prime}$(*) structure, in perfect agreement with the observations reported in ref. ^21^.

Changes in the spin splitting should also affect the response in the Weyl point area (ROI D). However, in this case, the situation is more complex as the area covers at the same time the signal from the close-lying and spin-split electron pocket and upper hole pocket (see Fig. 4c), which at low temperatures give rise to the formation of the Weyl points. A reduction (increase) in the spin splitting upon excitation of the shear mode will at the same time increase (reduce) the separation between electron pocket and hole pocket^27^. The anti-phase behavior of the latter process with respect to the spin splitting may explain why the PE signal from the Weyl point area is *π*-shifted in comparison to all other ROI.

Reference ^27^ finally provides also information on how the highest of the three bulk bands will be affected by a shear motion along the *b* axis. Due to the much smaller spin splitting of this band in comparison with the electron and hole pockets, the calculation predicts only very subtle changes even for a full phase transition. In the Fourier map, this band is indeed the only band which lacks a clear signal along its band dispersion. The two other bulk bands are in contrast not considered in the calculations in ref. ^27^. However, band structure calculations imply that these two bands show a distinct dispersion along the Γ–Z direction (Supplementary Note 11), i.e., in *c*-direction (see Fig. 1). It is therefore not surprising that particularly these bands become affected by a shear displacement among neighboring WTe_2_ layers giving rise to rather large amplitudes in the Fourier map.

The excitation of coherent phonons provides unique opportunities for the study and coherent control of structural, electronic, and magnetic properties of solids^34–36^. Time- and angle-resolved photoemission spectroscopy is in this context the most direct instrument to map the electronic structure response at the required energy- and momentum sensitivity^37–39^. The phonon mode-resolved Fourier maps introduced in the present work allow in a very direct and intuitive manner for an electron-band selective view onto electron–phonon interaction processes including even electronic states above *E*_F_. The striking differences observed among the Fourier maps of *Td*-WTe_2_ emphasize the band-selectivity of coherent phonon excitation processes. Remarkably, in the Fourier map representation, the nonlinear signal response to the excitation of coherent phonons can substantially enhance the spectral resolution uncovering spectral details not visible in the ARPES spectra.

Our results reveal, furthermore, that the excitation of a low-frequency interlayer shear mode periodically modulates the spin splitting of bands, a spectral signature that is closely linked to the broken inversion symmetry of the crystalline lattice. In addition, the data prove that the excitation of the shear mode affects the electronic structure in the energy-momentum area comprising the Weyl points in *Td*-WTe_2_ at low temperatures. Overall, the presented experimental results strongly support the relevance of the shear mode excitation for the control of the specific Weyl physics in this material as recently predicted in ref. ^21^.

## Methods

### Sample synthesis

High-quality *Td*-WTe_2_ crystals were grown using a chemical vapor transport technique. Stoichiometric tungsten powder (99.9%) and tellurium powder (99.99%) were ground together and loaded into a quartz tube with a small amount of the transport agent TeBr_4_. All weighing and mixing was carried out in a glove box. The tube was sealed under vacuum and placed in a two-zone furnace. The hot zone and the cold zone were maintained for 1 week at a constant temperature of 800 and 700 °C, respectively.

### TRARPES experiments

For the TRARPES experiments, we used two non-collinear optical parametric amplifiers (NOPAs) which are pumped by the second harmonic of a chirped pulse amplifier. One of the NOPAs delivers 1.5 eV (827 nm), 30 fs, p-polarized pump pulses with an incident fluence of 110 μJ cm^−2^ on the sample. The 840 nm output of the second NOPA system is used to generate 5.9 eV (210 nm), 95 fs, s-polarized probe pulses by sequential frequency doubling. Cross-correlation measurements at the sample position yielded  ≈100 fs FWHM (Supplementary Note 2). Right before the pump-probe experiments, the *Td*-WTe_2_ crystals were cleaved under ultra-high vacuum conditions. The samples were aligned by low-energy electron diffraction in a direction crossing $\overline{\Gamma }$ and the projection $\overline{{\rm{W}}}$ of the predicted position of two neighboring Weyl points onto the (001) surface (see Fig. 1a). ARPES spectra were recorded using a hemispherical analyzer at a total energy resolution of 40 meV. All experiments were performed at room temperature at a pressure of 2 × 10^−10^ mbar.

### Band structure calculations

The electronic bulk band structure was calculated by *ab-initio* calculation based on density functional theory with projector augmented-wave method^40^ as implemented in the Vienna Ab-initio Simulation Package (VASP)^41^. The exchange and correlation energies were considered on the level of the generalized gradient approximation with a Perdew–Burke–Ernzerhof functional^42^. The energy cutoff was set to be 350 eV for the plane wave basis. We included the van der Waals corrections via a pair-wise force field of the Grimme method^43^. The experimental lattice constants from ref. ^44^ were used in all the calculations.

## Supplementary information

Supplementary Information

## Data Availability

All data that support the findings of this study are available from the corresponding author upon reasonable request.
