# Supplementary material for: Mode-resolved reciprocal space mapping of electron-phonon interaction in the Weyl semimetal candidate Td-WTe2
Source: Nat Commun. 2020 May 26;11:2613. doi: 10.1038/s41467-020-16076-0 (PMC7250889; doi:10.1038/s41467-020-16076-0)
Supplement: Supplementary file 1 — Supplementary Information [file 41467_2020_16076_MOESM1_ESM.pdf]

Supplementary Material for
"Mode-resolved reciprocal space mapping of electron-phonon interaction in the Weyl semimetal candidate *Td*-WTe_2"

Petra Hein, Stephan Jauernik, Hermann Erk, Lexian Yang,
Yanpeng Qi, Yan Sun, Claudia Felser, and Michael Bauer

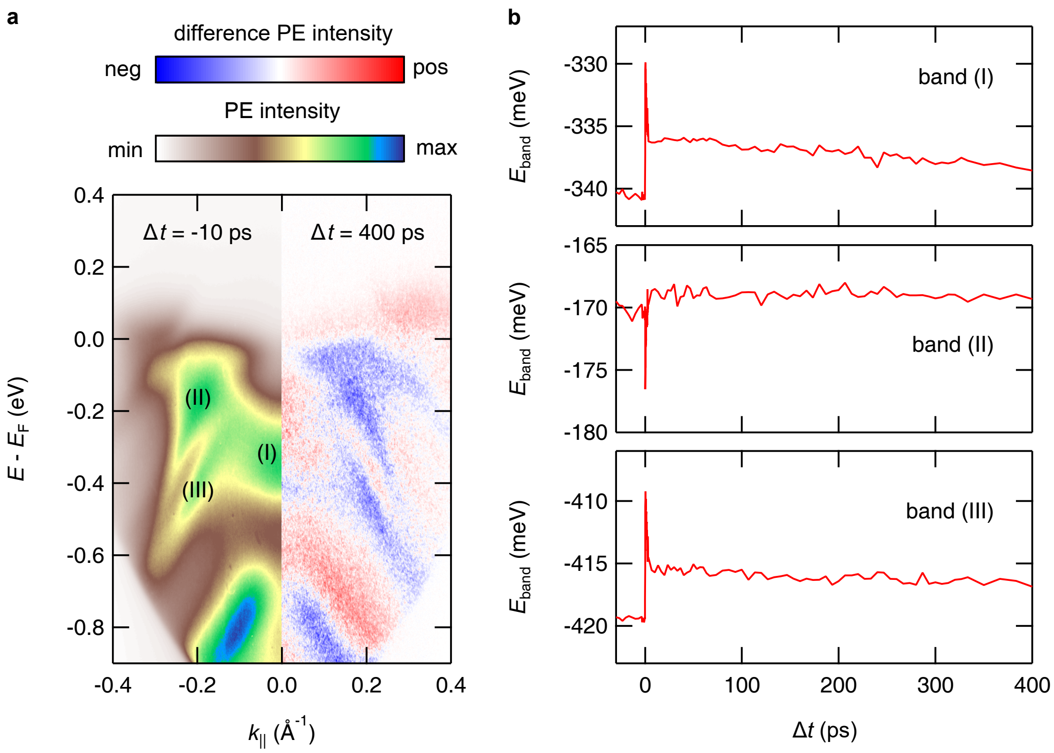

Supplementary Fig. 1. Long-lived spectral changes in the TRARPES data of Td-WTe_2. (**a**) ARPES intensity map recorded at $\Delta t = -10\,\text{ps}$ in comparison to a difference intensity map at $\Delta t = 400\,\text{ps}$. (**b**) Peak energies of the three bands indicated in (**a**) as a function of Δt.

SUPPLEMENTARY NOTE 1: LONG-LIVED SPECTRAL CHANGES

Time-resolved ARPES results covering an extended pump-probe delay range up to $\Delta t = 400\,\text{ps}$ are compiled in Supplementary Fig. 1. Supplementary Fig. 1a shows a ground state ARPES intensity map recorded 10 ps before the optical excitation in comparison to a difference intensity map at $\Delta t = 400\,\text{ps}$. The presence of red and blue areas in the difference intensity map indicates that at this time delay the system has still not reached its initial equilibrium state. To further illustrate the long-lived spectral changes, we analyzed the transient peak shifts of three bands labeled (I), (II), and (III) in Supplementary Fig. 1a. The peak positions are determined from Gaussian fits to the transient EDCs of the respective bands. The results of the analysis are summarized in Supplementary Fig. 1b. All bands exhibit rapid energy shifts between 7 meV and 11 meV at time zero followed by an exponential relaxation on a few picosecond time scale. We assign these dynamics to band renormalization processes in response to the excitation and subsequent relaxation of hot carriers. At

$\Delta t \approx 5\,\mathrm{ps}$, the energy of band (II) has relaxed back to nearly its initial value, while bands (I) and (III) still exhibit clear energy offsets of $\approx 4\,\mathrm{meV}$. At the maximum time delay of 400 ps, the two bands still show offsets of $\approx 2\,\mathrm{meV}$. Other time-resolved studies reported on similar long-lived spectral changes in Td-WTe_2 [1, 2]. Their persistence was associated with heat diffusion out of the excitation volume on a characteristic time scale of several nanoseconds [2].

SUPPLEMENTARY NOTE 2: TIME RESOLUTION AND SAMPLING FREQUENCIES

The coherent phonon frequency range accessible in our TRARPES experiments depends on two factors: the time resolution of the experiment and the sampling rate used for the data acquisition. We determined the time resolution from a pump-probe cross-correlation measurement of a polycrystalline gold film. The cross-correlation trace is shown in Supplementary Fig. 2a. It was extracted from TRARPES data for an electron binding energy of $E - E_\mathrm{F} = 1.3\,\mathrm{eV}$ in order to keep the broadening due to finite intermediate state lifetimes small [3]. A Gaussian fit to the trace yields a FWHM of $\approx 100\,\mathrm{fs}$ for the time resolution of our setup.

In our TRARPES experiments, we measure in the time domain the convolution of the sample response (i.e., the coherent phonon-induced oscillations) and the cross-correlation trace representing the finite time resolution. In Fourier space, i.e., in the frequency domain, this corresponds to the product of the Fourier spectrum of the sample response and the Fourier spectrum of the cross-correlation trace. Hence, the latter can be considered as a spectral transmission function determining the amplitude at which a phonon peak is probed in the experiment. The Fourier spectrum of the Gaussian fit to the experimental cross-correlation trace is shown in Supplementary Fig. 2b. Markers indicate the frequencies of the five coherent phonon modes observed in our measurements. Due to the frequency dependence of the transmission function, the detected signal amplitude of the highest frequency mode at 6.35 THz becomes weakened by a factor of about 4 in comparison to the lowest frequency mode at 0.23 THz. However, all five modes are probed at a reasonable amplitude. Even more, also the highest energy optical phonon mode of Td-WTe_2 with a frequency of approximately 7.2 THz [4, 5] would be covered reasonably well by the spectral transmission

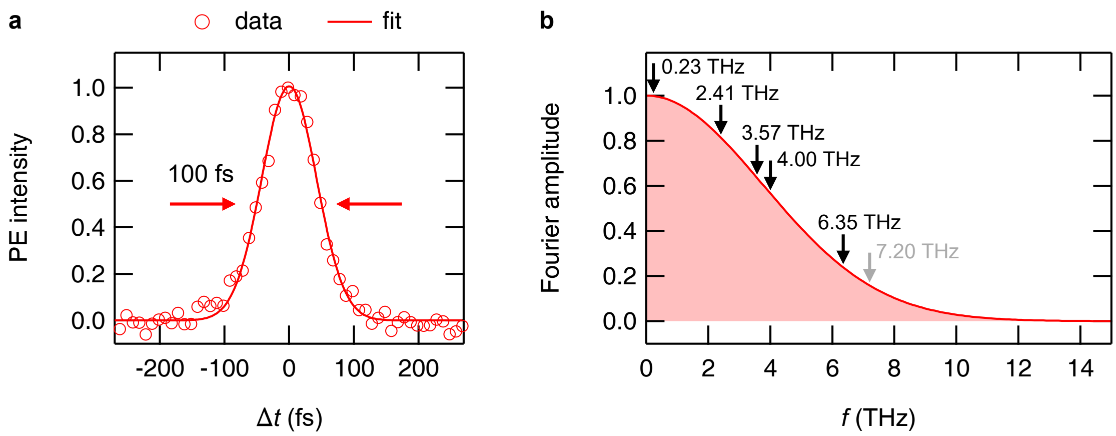

Supplementary Fig. 2. Time resolution of the TRARPES experiment. (**a**) Experimental NIR-pump/NUV-probe cross-correlation trace measured via photoemission from a polycrystalline gold film at $E - E_F = 1.3\,\text{eV}$. The solid line is a Gaussian fit to the experimental data. (**b**) Fourier spectrum of the Gaussian fit in (**a**). The black markers indicate the coherent phonon frequencies observed in the TRARPES data of Td-WTe_2. The grey marker indicates the highest frequency optical phonon mode of Td-WTe_2.

function. In summary, the available time resolution of the experiment in principle allows for the real-time detection of optical phonons within the entire phonon spectrum supported by Td-WTe_2. For quantitative conclusions on the excitation amplitudes of different phonon modes one should, however, keep in mind that the sensitivity of the experiment decreases with increasing frequency.

Independently of the transmission function resulting from the finite time resolution, the accessible frequency range of the experiment also depends on the sampling rate used for the data acquisition and the overall delay range of the measurement. Based on the Nyquist sampling criterion, the sampling frequency must be greater than twice the maximum frequency contained in the signal to be able to identify all frequency components correctly via Fourier transformation. Considering the highest energy phonon branch in Td-WTe_2 with a frequency of approximately 7.2 THz [4, 5], we therefore chose a minimum sampling rate of 15 THz for our measurements.

For a fixed number of data points, increasing the sampling frequency improves the accuracy at which the time-domain signal is traced. At the same time, it worsens the frequency

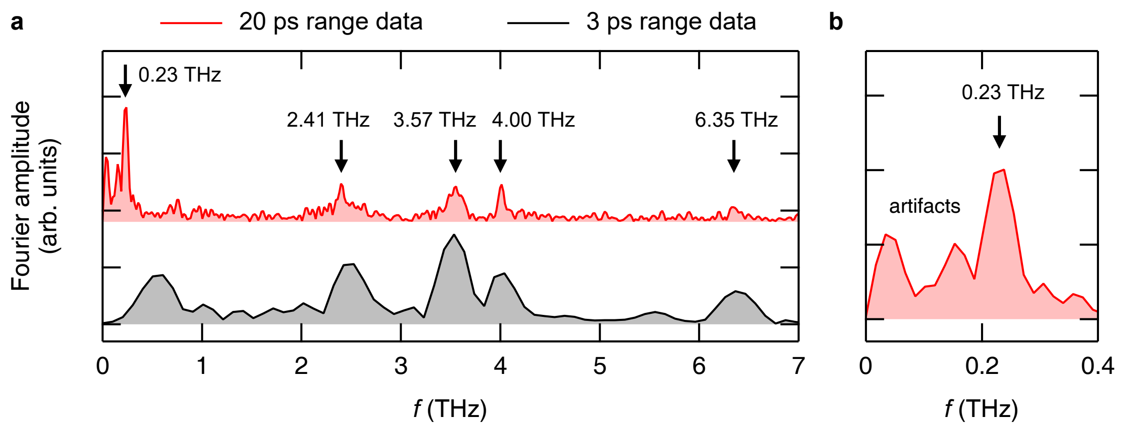

Supplementary Fig. 3. Fourier amplitude spectra of the PE intensity transients shown in Figs. 2c and 2d of the main text. (**a**) Comparison of the Fourier amplitude spectra of the 3 ps range data and the 20 ps range data. (**b**) Close-up of the low-frequency range of the 20 ps range data.

resolution of the Fourier transformation that is given by the reciprocal total sampling time, i.e., the overall delay range chosen in the experiment. To be able to track the high- as well as the low-frequency coherent phonon-induced oscillations in the time domain, we recorded TRARPES data sets using two different sampling rates: A sampling rate of 60 THz enabled us to measure the beating signal caused by the superposition of coherent phonon modes with frequencies between 2.41 THz ($T = 415$ fs) and 6.35 THz ($T = 157$ fs) (cf. Fig. 2c in the main text). For these measurements, the total delay range was restricted to 3 ps to keep the overall data acquisition time at an acceptable level. For the low-frequency interlayer shear mode ($T = 4.35$ ps), we chose a significantly longer delay range of 20 ps using a sampling rate of 15 THz to still comply with the Nyquist sampling criterion (cf. Fig. 2d in the main text). Note that similar to conventional frequency domain spectroscopy, it is allowed and (even more) convenient to choose delay steps for accurately probing the time-domain signal that are significantly smaller than the time resolution - in the present study delay steps of 17 fs and 67 fs at a time resolution of 100 fs, respectively.

Supplementary Fig. 3a compares the Fourier amplitude spectra of the 3 ps and 20 ps range intensity transients shown in Figs. 2c and 2d of the main text. The frequency increment of the Fourier analysis, given by the reciprocal total sampling time, is ≈ 0.3 THz and ≈ 0.05 THz for the 3 ps and 20 ps range data, respectively. While the four high-frequency components

are already evident in the amplitude spectrum of the 3 ps range data, their shape becomes much clearer in the amplitude spectrum of the 20 ps range data allowing for a more precise determination of the peak positions as expected from the discussion above for an extended overall delay range. The 0.23 THz interlayer shear mode can only be resolved in the 20 ps range data set. Furthermore, we observe additional peaks at frequencies below 0.23 THz (see Supplementary Fig. 3b). They do not match any of the predicted or observed phonon modes of Td-WTe_2 [2, 4–7]. In contrast to the five main frequency peaks between 0.23 THz and 6.35 THz, the positions and relative heights of these very-low frequency peaks are quite sensitive to the data analysis procedures, e.g., the delay range included in the analysis and the model function used for background subtraction. We therefore conclude that the appearance of these peaks arises from artifacts of the data analysis.

SUPPLEMENTARY NOTE 3: BACKGROUND CORRECTION OF PHOTOEMISSION TRANSIENTS

The temporal evolution of the transient PE signal of Td-WTe_2 is mainly determined by contributions from the hot carrier dynamics and the coherent phonon oscillations. To separate the two signal contributions, we fitted the PE intensity transients with a model function accounting for the carrier population dynamics. Subtraction of the fitting result from the experimental data enables us to extract the pure oscillatory part of the signal. For the fits, we used the following function:

$$I(\Delta t) = \begin{cases} I_{01}, & \Delta t < t_0 \\ I_{02} + A_1 \cdot \exp\left(-\frac{\Delta t - t_0}{\tau_1}\right) + A_2 \cdot \exp\left(-\frac{\Delta t - t_0}{\tau_2}\right), & \Delta t \geq t_0 \end{cases} \quad (1)$$

Here, I_{01} accounts for the constant PE intensity before photoexcitation ($\Delta t < t_0$) and I_{02} accounts for an intensity offset at the maximum investigated delay caused by long-lived spectral changes. At the time t_0, excited carrier population and depopulation dynamics set in. They are modeled using a sum of two exponential functions describing an exponential rise ($A_1 < 0$) and decay ($A_2 > 0$) of the transient PE intensity with time constants τ_1 and τ_2, respectively. To account for the time resolution Δt_{FWHM} of the experiment, the fitting function was convolved with a Gaussian function $g(t)$:

$$g(t) = \exp\left(-\frac{4 \cdot \ln 2 \cdot t^2}{{\Delta t_{\mathrm{FWHM}}}^2}\right). \quad (2)$$

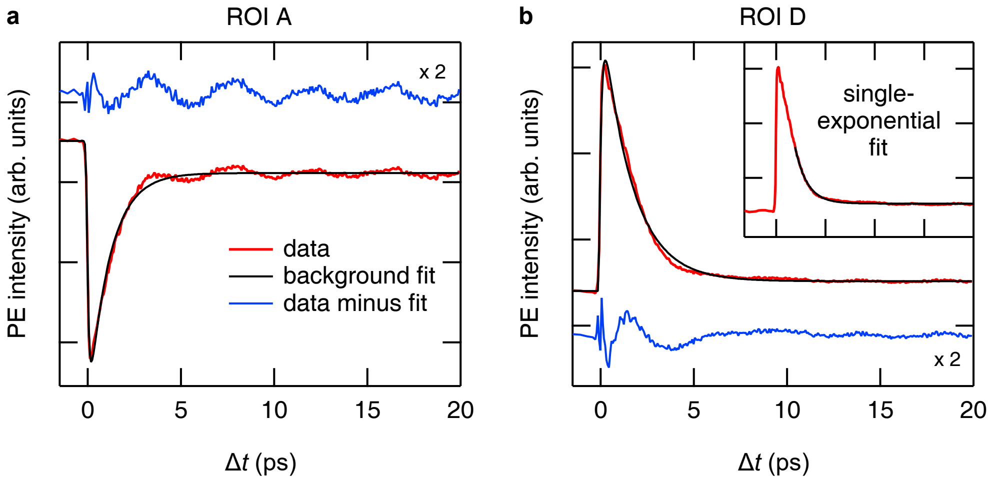

Supplementary Fig. 4. Subtraction of hot carrier contribution. (**a**) PE intensity transient from ROI A (red) including a fit to the data starting at $\Delta t = -1.5\,\mathrm{ps}$ according to Eq. (1) (black line). The blue line shows the PE intensity transient after subtraction of the fitting result. (**b**) Same as (**a**) for ROI D. The inset shows the same PE intensity transient including a single exponential fit starting at $\Delta t = 2\,\mathrm{ps}$.

Analog fit functions were used to account for the hot carrier response in the peak energy $E_{\mathrm{band}}(\Delta t)$ and peak width $\Delta E(\Delta t)$ transients.

For most cases, Eq. (1) is appropriate to account for the dynamics associated with the hot carrier processes. As an example, Supplementary Fig. 4a shows the PE intensity transient for ROI A in Fig. 4a of the main text and the corresponding fit using Eq. (1). However, in regions near E_{F}, we observed deviations from a pure exponential signal decay, including the Weyl point region (ROI D, cf. Fig. 4d of the main text). The PE intensity transient from this region is shown in Supplementary Fig. 4b including the result of a fit using Eq. (1). The deviation from an exponential decay is clearly visible and results in a signal distortion at small delays in the hot carrier-corrected data shown as blue line. The quality of the background subtraction procedure can be improved for this type of data by restricting the delay range to $\Delta t > 2\,\mathrm{ps}$ and by describing the hot carrier processes with a single exponential fit function. Still, a complete removal of hot carrier contributions is not possible resulting in a residual signal visible as a distortion in Fig. 4d between $\Delta t = 5\,\mathrm{ps}$ and $\Delta t = 10\,\mathrm{ps}$.

SUPPLEMENTARY NOTE 4: COMPILATION OF FOURIER MAPS

For the energy- and momentum-resolved Fourier analysis of the data, the ARPES intensity maps were divided into small integration regions with a size of $23\,\mathrm{meV} \times 0.01\,\mathrm{\AA}^{-1}$ (Figs. 3a and 4a) or $6\,\mathrm{meV} \times 0.0025\,\mathrm{\AA}^{-1}$ (Fig. 5a). Each of these regions features a PE intensity transient showing a superposition of carrier population dynamics and coherent phonon-induced oscillations. Some spectral regions show an additional signal confined to the delay range around time zero (cf. Fig. 4d and 4e of the main text) that we assign to the pump-probe laser cross-correlation. We therefore restricted the data range for the analysis to a time delay range starting well after this signal contribution. Before the Fourier transformation, the PE intensity transients from each region were fitted for background subtraction with a single-exponential function to account for the carrier relaxation dynamics. The 3 ps range data were analyzed for a delay range starting at $\Delta t = 280\,\mathrm{fs}$. In the case of the 20 ps range data, we had to exclude the first 2 ps after time zero as a single-exponential fit of the intensity transients especially around the Fermi level results otherwise in low-frequency artifacts that interfere with the 0.23 THz oscillation (see intensity transient of ROI D shown in Supplementary Fig. 4b).

After subtraction of the single-exponential background, the resulting transients were zero-padded to triple their lengths before their amplitude spectra were determined via fast Fourier transformation. This results in an energy- and momentum-dependent data set of Fourier amplitude spectra. A cut through this data set at a fixed frequency represents the energy- and momentum-resolved Fourier amplitude at the selected frequency. It can be displayed as a color-coded image which we refer to as Fourier map. Phonon-mode selectivity is achieved by selecting the frequencies corresponding to the coherent phonon modes. As an extended version of Fig. 3a in the main text, Supplementary Fig. 5 shows Fourier maps at 0.23 THz, 2.41 THz, 3.57 THz, 4.00 THz, and 6.35 THz in comparison to the excited state ARPES intensity map at $\Delta t = 120\,\mathrm{fs}$. The 0.23 THz Fourier map results from the analysis of the 20 ps range data. In contrast, the four high-frequency Fourier maps are derived from the 3 ps range data.

We finally would like to add that the details of the Fourier map patterns critically depend on the type of spectral changes in the ARPES data that are induced by the coherent phonon excitation. In general, a pure periodic modulation in the PE intensity results in a slowly

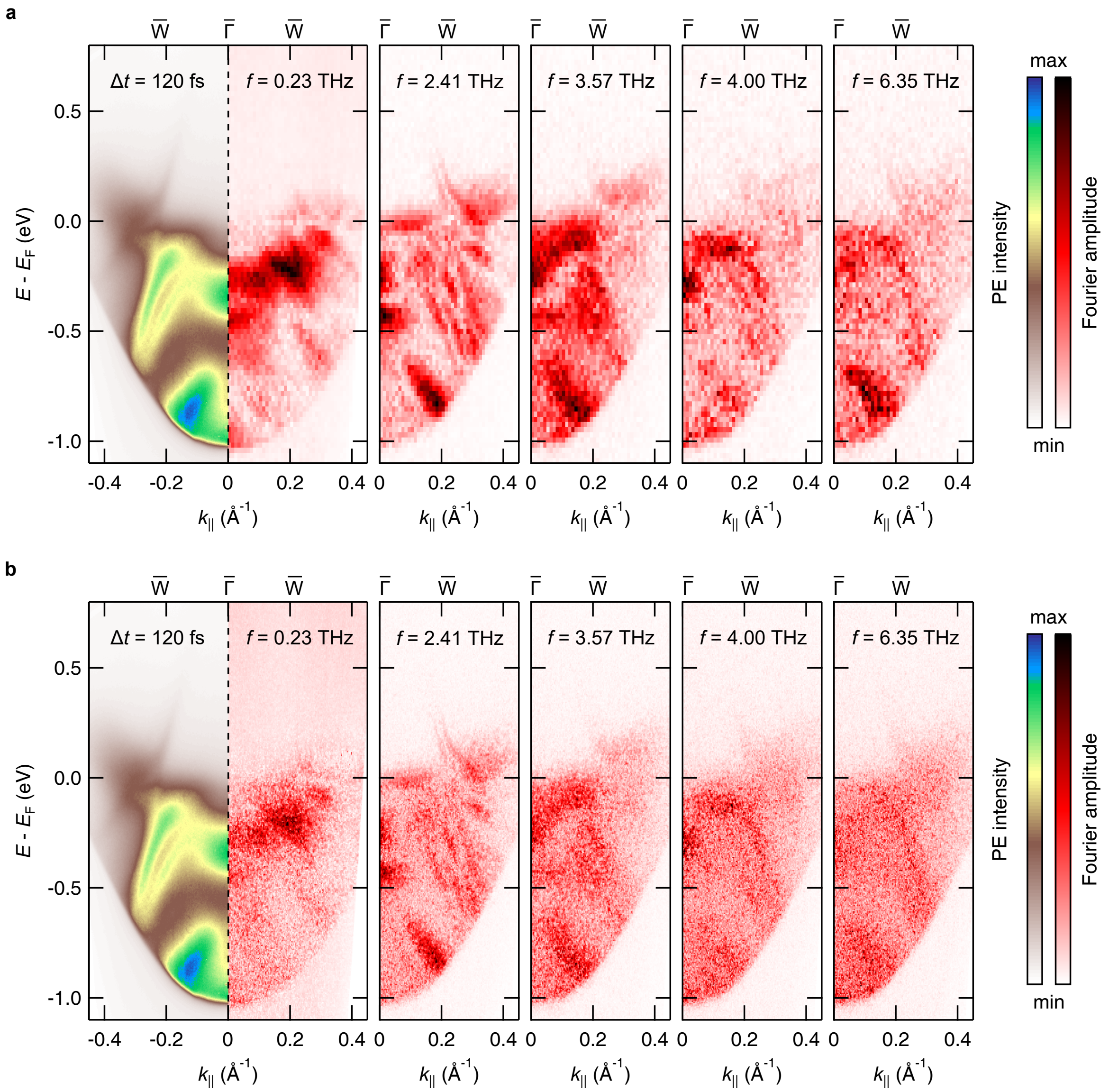

Supplementary Fig. 5. Phonon mode-selective Fourier amplitude analysis of the TRARPES data. ARPES intensity map at $\Delta t = 120\,\mathrm{fs}$ in comparison to Fourier maps at the five phonon frequencies identified in the Fourier amplitude spectra. (**a**) Fourier maps generated from ARPES intensity maps that were binned over regions of $23\,\mathrm{meV} \times 0.01\,\text{Å}^{-1}$. (**b**) Fourier maps generated from ARPES intensity maps that were binned over regions of $6\,\mathrm{meV} \times 0.0025\,\text{Å}^{-1}$.

varying Fourier amplitude in the energy and momentum range of the signal modulations. In contrast, changes in peak energy or peak width result in distinct minima in the Fourier amplitude right at the peak position and Fourier amplitude maxima on both sides next to the peak. These effects are best seen when comparing the 0.23 THz and 2.41 THz Fourier maps.

As discussed in the main text, the oscillations in the 0.23 THz data predominantly arise from PE intensity modulations resulting in a rather smooth Fourier map pattern. In contrast, the 2.41 THz Fourier amplitude shows a distinct fine structure of amplitude mimima that are indicative for shifts in the band energies or changes in the band widths in response to the excitation of the 2.41 THz phonon mode.

SUPPLEMENTARY NOTE 5: RESOLUTION ENHANCEMENT

The effect of resolution enhancement in the Fourier maps is illustrated in Supplementary Fig. 6. Supplementary Fig. 6a shows a close-up of the ARPES intensity map in Fig. 3a of the main text with a selected energy-momentum cut indicated by the grey-shaded bar. The EDC along this cut (Supplementary Fig. 6c) exhibits three peaks with widths in the order of 100 meV governed by the experimental resolution of 40 meV and considerable thermal broadening due to the sample temperature of 300 K. Supplementary Fig. 6b shows for comparison the corresponding 2.41 THz Fourier map exhibiting a distinct fine structure not visible in the ARPES intensity map. The data is overlaid with experimental band dispersions from a high-resolution ARPES study conducted with an energy resolution of 2 meV and at a sample temperature below 20 K [8]. In the overlapping energy-momentum regime, the experimental band dispersions exhibit a striking agreement with the dispersion of the fine structure. Even more, an intuitive extrapolation of part of the data from Ref. [8] to higher binding energies also seems to follow the fine structure in the 2.41 THz Fourier map. Supplementary Fig. 6d displays the Fourier amplitude as a function of binding energy along the selected energy-momentum cut, in which the fine structures appears as distinct and narrow amplitude dips. The spectral width of these dips is typically in the order of 20 meV and, therefore, much smaller than the width of the characteristic spectral signatures in the ARPES intensity maps and also smaller than the spectral resolution of our experiment. Along the selected energy-momentum cut, we identify in the Fourier amplitude signal five dips in comparison to three peaks in the EDC, implying that the obvious resolution enhancement allows observing details that are hidden in the ARPES data due to strong spectral broadening. The assignment of the dips to different bands is indicated by numbers (cf. Supplementary Fig. 6b). Bands number 1, 2, and 4 were assigned based on an extrapolation of the high resolution ARPES data to higher binding energies, while the assignment of bands number 3 and 5 results from

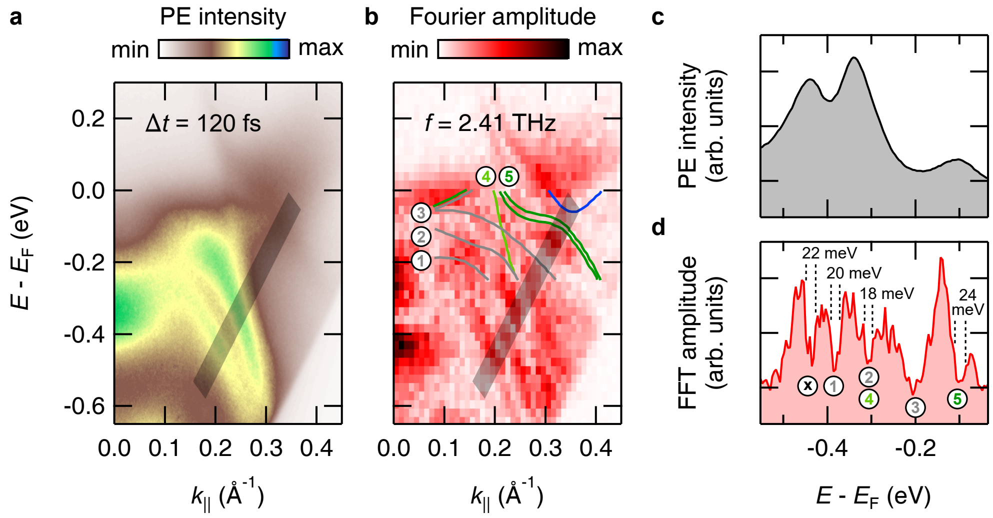

Supplementary Fig. 6. Resolution enhancement in the Fourier maps. Close-ups of (**a**) the ARPES intensity map and (**b**) the 2.41 THz Fourier map shown in Fig. 3a of the main text with the energy-momentum cut analyzed in (**c**) and (**d**) indicated. The Fourier map was generated from ARPES intensity maps that were binned over regions of 23 meV $\times$ 0.01 $Å^{-1}$. It is overlaid with experimental band dispersions from Ref. [8]. Surface bands were omitted for the sake of clarity. (**c**) Energy distribution curve extracted from the ARPES intensity map along the energy-momentum cut indicated in (**a**). (**d**) Fourier amplitude as a function of binding energy along the energy-momentum cut indicated in (**b**). The data were extracted from a Fourier map generated from ARPES data that were binned over regions of 6 meV $\times$ 0.0025 $Å^{-1}$. The numbers indicate the assignment of the Fourier amplitude dips to the different bands shown in (**b**). 'x' indicates a band signature visible in the Fourier map that is outside the energy range that was probed in Ref. [8]. Spectral widths of selected dips are indicated.

the direct comparison of the Fourier map with the band dispersions. The amplitude dip labeled 'x' at $E - E_\mathrm{F} \approx -0.45$ eV could not be assigned to any of the indicated bands as the related fine structure is completely outside the energy range probed in Ref. [8]. We assume, however, that also this fine structure follows the dispersion of an electronic band of Td-WTe_2.

The origin of the resolution enhancement in the Fourier maps is schematically illustrated by a simulation presented in Supplementary Fig. 7. We considered a Gaussian peak with a width of 40 meV (FWHM) (Supplementary Fig. 7b) that is subject to a periodic peak energy

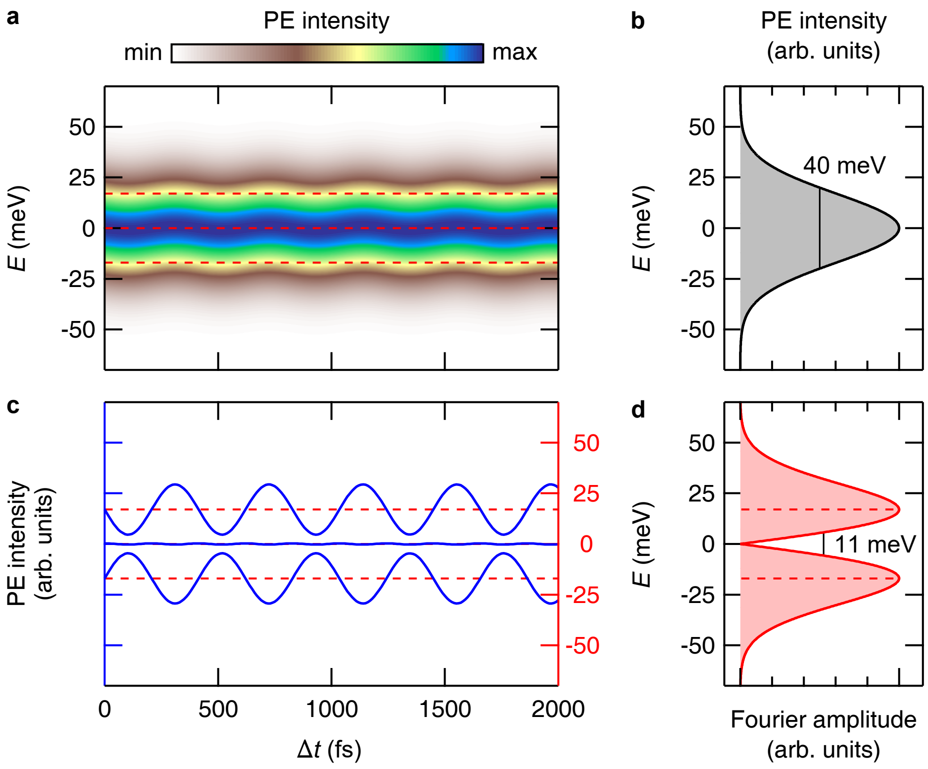

Supplementary Fig. 7. Origin of resolution enhancement. (**a**) Simulated PE intensity transient of the Gaussian peak shown in (**b**). The peak energy is periodically modulated with an amplitude of 1 meV and a frequency of 2.41 THz. (**c**) Changes in the PE intensity as a function of Δt (blue lines) at three selected energies indicated by the dashed red lines. The dashed red lines also indicate the respective zero of the changes in the PE intensity. (**d**) 2.41 THz Fourier amplitude as a function of energy determined from a Fourier transformation of the PE intensity transient shown in (**a**).

shift with an amplitude of 1 meV and a frequency of 2.41 THz (Supplementary Fig. 7a). A quantitative analysis of the oscillation amplitude shows that near the peak maximum the changes in the PE intensity are considerably smaller than in large parts of the flanks of the Gaussian peak (Supplementary Fig. 7c). This energy dependence directly translates into the 2.41 THz Fourier amplitude signal, which is shown in Supplementary Fig. 7d. At the energy of the peak maximum, the Fourier amplitude exhibits a distinct dip with a width (FWHM) that is significantly smaller than the width of the oscillating PE signal. In the present example, we observe in the Fourier signal in comparison to the PE signal a reduction of the width by a factor of ≈ 4 to a value of 11 meV. Note, however, that the situation is in general more complex as different dynamical processes such as peak shift, peak broadening,

and an overall modulation of spectral weight can independently superimpose each other.

SUPPLEMENTARY NOTE 6: TIME-DEPENDENT FOURIER AMPLITUDE AND PHASE ANALYSIS

A time-dependent Fourier amplitude analysis enables us to gain information on the lifetime of the different coherent phonons and to identify possible mode frequency shifts as a function of time. Furthermore, a time-dependent Fourier phase analysis of phonon-induced band shifts allows for conclusions on the underlying excitation mechanisms of the coherent phonons, i.e., the distinction between a displacive excitation (DECP) scenario [9] and an impulsive stimulated Raman scattering (ISRS) excitation scenario [10].

Supplementary Fig. 8 shows results of a time-dependent amplitude and phase analysis of the band energy transient of the prominent feature centered at $\bar{\Gamma}$ and $E - E_\mathrm{F} \approx -0.3\,\mathrm{eV}$ in the ARPES intensity maps. The data comprise the frequency range of the four high-frequency coherent phonon modes, which periodically modulate the peak energy as discussed in the main text (cf. Figs. 3b and 3c). For a time-dependent analysis, we successively shortened the band energy transient shown in Fig. 3b of the main text at its beginning and performed a Fourier transformation of the remaining transient, respectively. Supplementary Fig. 8a displays the resulting Fourier amplitude as a function of frequency and starting point τ of the analyzed transient. The results illustrate the distinct differences in the lifetimes of the different modes, which obviously also conform with their differences in spectral width. The 3.57 THz mode and the 6.35 THz mode become damped within less than 6 ps, whereas the 2.41 THz mode and the 4.00 THz mode are still visible at 12 ps. For the 2.41 THz mode, we additionally observe a fast decaying shoulder which may hint to the excitation of a neighboring short-living coherent phonon. The data do not show any indications for a spectral shift of the modes as a function of time.

Supplementary Fig. 8b shows the Fourier amplitude spectrum for $\tau = 0\,\mathrm{fs}$ extracted from Supplementary Fig. 8a. We used this spectrum to determine the positions of the peak maxima (indicated by the arrows) for which we performed a time-dependent Fourier phase analysis. The results of the phase analysis are shown in Supplementary Fig. 8c. We additionally performed a phase analysis for the data points next to the positions of the peak maxima at $\pm 0.016\,\mathrm{THz}$ to estimate the error in the determined phase. All results are

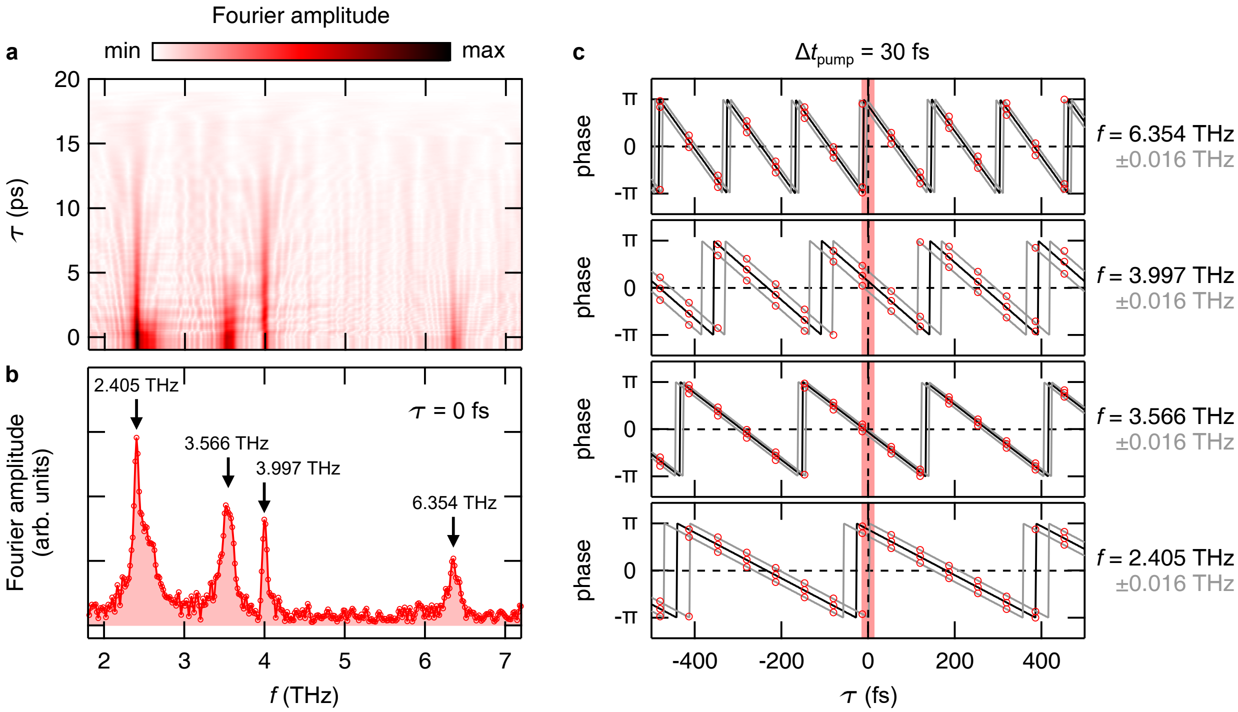

Supplementary Fig. 8. Time-resolved Fourier amplitude and phase analysis of the high-frequency modes. (**a**) Fourier amplitude as a function of frequency and starting point τ of the band energy transient shown in Fig. 3b of the main text. Details on the evaluation of the data are given in the text. (**b**) Fourier amplitude spectrum extracted from (**a**) for $\tau = 0\,\text{fs}$. (**c**) Fourier phase as a function of τ for the peak positions of the four high-frequency modes. The black and gray lines show fits of a sawtooth function to the peak maximum data and the data of neighboring frequencies, respectively. The red shaded area at $\tau = 0\,\text{fs}$ indicates the uncertainty in determining the time zero of the experiment due to the temporal width of the pump pulse.

fitted with sawtooth functions with the amplitudes set to a value of π. The phases of the oscillations at time zero indicate the underlying phonon excitation mechanism. Time zero was determined from the peak position of the laser cross-correlation signal that is in part of the data visible during the temporal overlap of pump and probe pulse (cf. Fig. 4d and 4e in the main text). Our analysis yields initial phases of $(0.87 \pm 0.14)\pi$ for the excitation of the 2.41 THz mode, $(-0.07 \pm 0.06)\pi$ for the excitation of the 3.57 THz mode, $(0.14 \pm 0.22)\pi$ for the excitation of the 4.00 THz mode, and $(0.85 \pm 0.12)\pi$ for the excitation of the 6.35 THz mode. Within the error bars and under consideration of the pump pulse width, these results are compatible with a cosinusoidal response of the band energies to the excitation of the

coherent phonons implying for all cases a displacive excitation [9].

For the phase analysis of the low-frequency interlayer shear mode, we analyzed the band energy transient of ROI A′ as shown in Fig. 4e of the main text. In this case, the initial phase can be directly determined from a fit of the transient. Although overlaid with a laser cross-correlation PE signal around time zero, the oscillation can be clearly identified as sinusoidal (cf. black line in Fig. 4e of the main text), implying for this coherent phonon mode an impulsive excitation [10].

SUPPLEMENTARY NOTE 7: ESTIMATION OF THE SHEAR MODE AMPLITUDE

An approximate estimation of the shear mode amplitude in Td-WTe_2 resulting from the excitation with 827 nm pump pulses at an incident fluence of 110 µJ cm^{-2} becomes possible under consideration of quantitative results on the shear displacement reported for excitation with 23 THz pulses in Ref. [1]. To account for the different excitation wavelengths, we first referenced our data to fluence-dependent data on the shear mode frequency reported in a TRR study of Td-WTe_2 using 800 nm pump pulses [7]. Corresponding data for THz excitation as a function of the incident total pulse energy are also found in in Ref. [1]. Both studies report on an overall shift of the shear mode frequency of approximately 16 % over the probed pump fluence and energy ranges, respectively. Comparison of the data sets allow, therefore, to reference the 800 nm fluence values to the 23 THz pulse energy values. This comparison yields that the excitation with 800 nm, 150 µJ cm^{-2} pump pulses results in a shear amplitude approximately corresponding to the excitation with 23 THz pump pulses with a field amplitude of 0.65 MV cm^{-1}. Based on the quantitative discussion on the shear displacement in Ref. [1], this allows to roughly estimate the shear mode amplitude in our experiment to a value in the range of 1 pm. In this context, it is noteworthy that in Ref. [1] a complete annihilation of the Weyl points is predicted already for a shear displacement of 2 pm, even though the complete transition into the centrosymmetric $1T'(^*)$ phase requires an overall shear displacement of ≈ 12 pm.

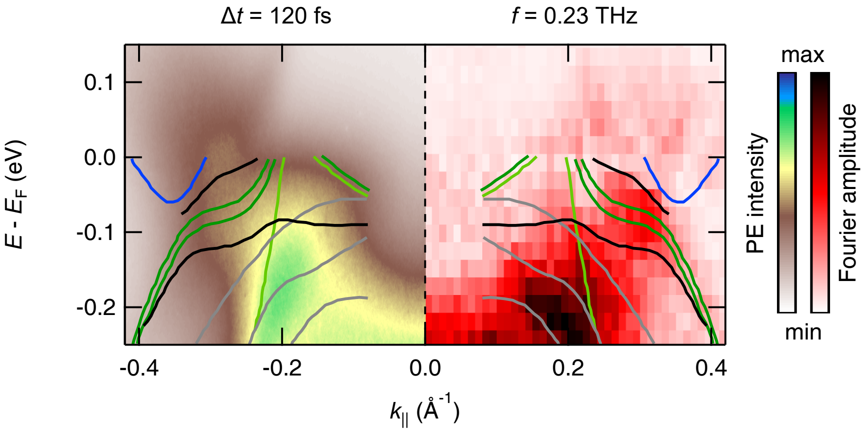

Supplementary Fig. 9. Close-ups of the ARPES intensity map near E_{F} at $\Delta t = 120\,\mathrm{fs}$ (left) and the corresponding 0.23 THz Fourier map (right). The data is overlaid with experimental band dispersions from Ref. [8]. In contrast to Fig. 4a of the main text, surface bands are included in black.

SUPPLEMENTARY NOTE 8: COMPARISON OF THE 0.23 THz DATA WITH BAND DISPERSIONS INCLUDING SURFACE BANDS

Supplementary Fig. 9 shows close-ups of an ARPES intensity map at $\Delta t = 120\,\mathrm{fs}$ and the corresponding 0.23 THz Fourier map overlaid with experimental band dispersions from Ref. [8] with the surface bands included.

SUPPLEMENTARY NOTE 9: COMPARISON OF ROI ABOVE E_{F}

Supplementary Fig. 10 compares PE intensity transients from the two energy-momentum areas above E_{F} that exhibit distinct amplitude maxima in the 0.23 THz Fourier map. In Supplementary Fig. 10a, the two areas (ROI D and ROI D′) are indicated in the Fourier map, with ROI D already introduced as the Weyl point area in the main text. The corresponding PE intensity transients are shown in Supplementary Fig. 10b. Whereas the signal from ROI D′ oscillates in phase with the signal from the ROI below E_{F}, ROI D shows a characteristic anti-phase behavior as already discussed in the main text.

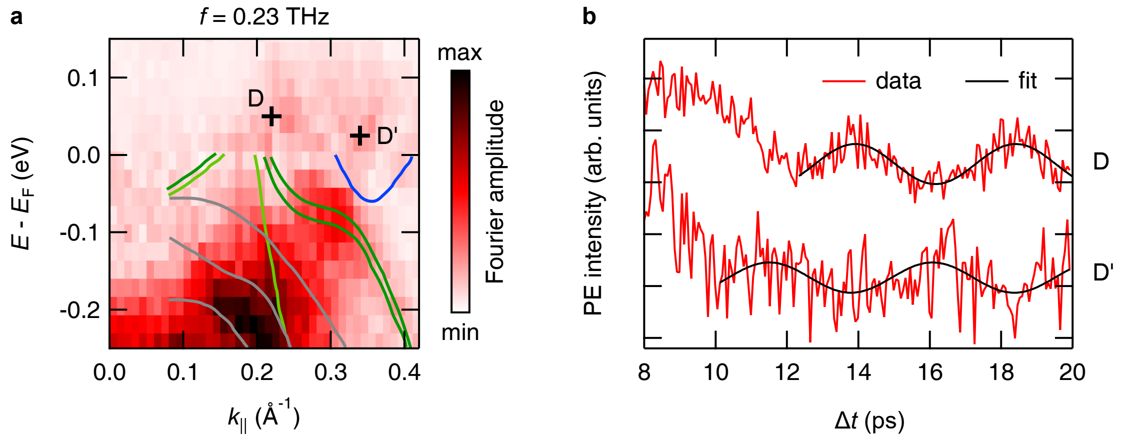

Supplementary Fig. 10. PE intensity transients from energy-momentum areas above E_F. (**a**) 0.23 THz Fourier map with ROI D and ROI D′ indicated. (**b**) Comparison of PE intensity transients from ROI D and ROI D′. The black lines are fits of a sinusoidal function to the data. For the analysis, we chose a signal integration area of 50 meV $\times$ 0.025 Å^{-1}.

SUPPLEMENTARY NOTE 10: SIMULATION OF THE FOURIER AMPLITUDE MODULATION OBSERVED IN ROI C

The procedure used to simulate the 0.23 THz Fourier amplitude modulation of the upper hole pocket observed near ROI C including the values chosen for the free parameters is schematically illustrated in Supplementary Fig. 11. The spin splitting of the bands was modeled by two Gaussians separated by 15 meV in accordance with results of high resolution ARPES data [8]. The peak width (FWHM) was set to 10 meV, simulations with different values showed, however, that this parameter does not critically affect our results. For modeling the dynamical response, we assumed that peak energies and peak intensities are modulated independently by the excitation of the shear mode. Peak energy shifts can result from band renormalization effects due to electron-phonon interaction as the crystal structure is periodically modulated by the excitation of a coherent phonon. Changes in the peak intensity (as detected in a photoemission experiment) can arise from changes in transition matrix elements, which are for instance sensitive to the crystal symmetry [11]. Oscillation amplitudes for rigid band shift [scenario (i)] and changes in the spin splitting [scenario (ii) and (iii)] were set to 1 meV. This value was estimated from the $\approx$ 15 meV equilibrium spin splitting of the bands [8] and under consideration of the approximate shear

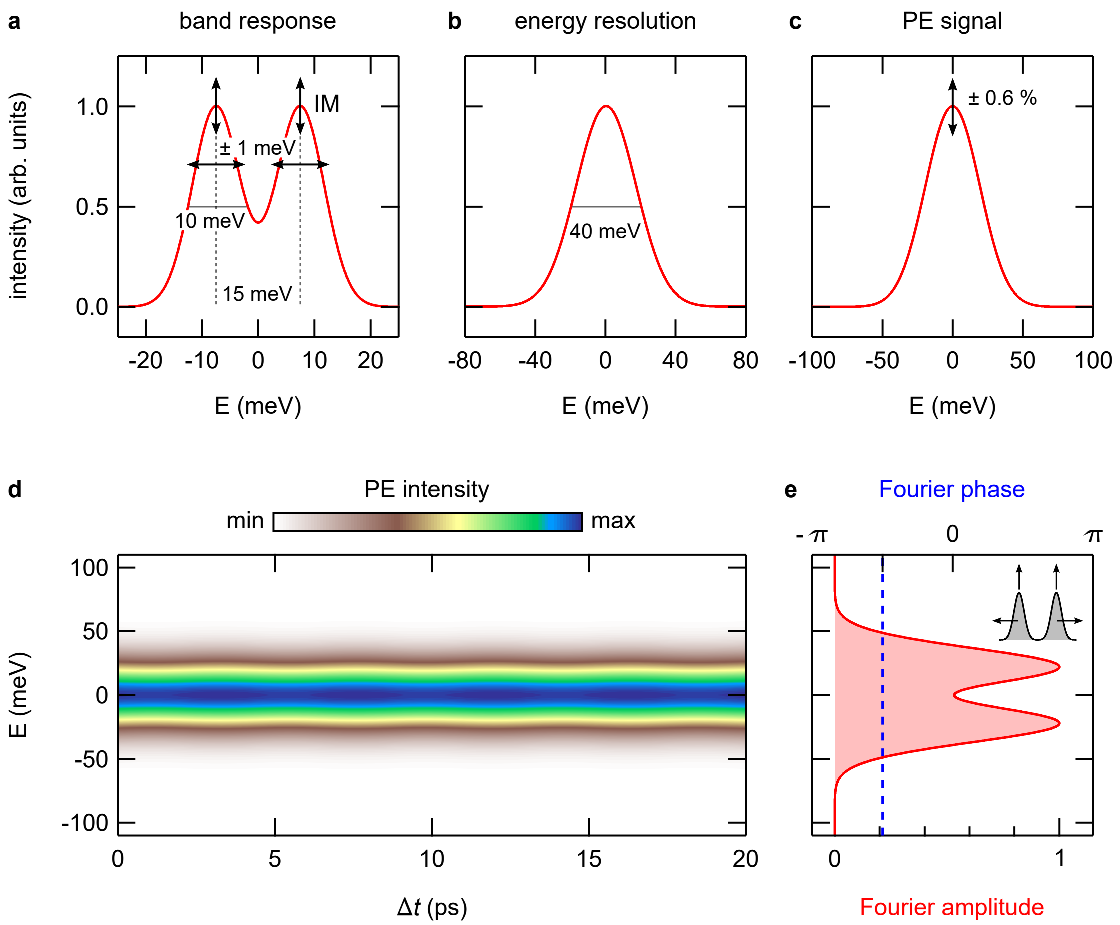

Supplementary Fig. 11. Schematic illustration of the procedure used to simulate the 0.23 THz Fourier amplitude modulation of the upper hole pocket. The spin splitting of the bands was modelled by two Gaussians (**a**), which were convolved by a Gaussian representing the energy resolution of the TRARPES experiment (**b**), yielding the PE signal (**c**). The parameters chosen for the simulations are indicated. The amplitude of the peak intensity modulation (IM) was adjusted until the resulting PE signal yielded a relative oscillation amplitude of $\pm 0.6\,\%$. (**d**) Simulated PE intensity transient for scenario (ii). The corresponding energy-dependent Fourier amplitude and phase signals (**e**) at the modulation frequency of 0.23 THz were determined from a Fourier transformation of the PE intensity transient (**d**).

mode amplitude of 1 pm at the applied excitation density (see Supplementary Note 7) with the latter value corresponding to $\approx 10\,\%$ of the shear displacement that transforms the Td structure into the centrosymmetric $1\,T'(^{*})$ structure [1]. To account for the energy resolution of the experiment, the band response (Supplementary Fig. 11a) was convolved with a

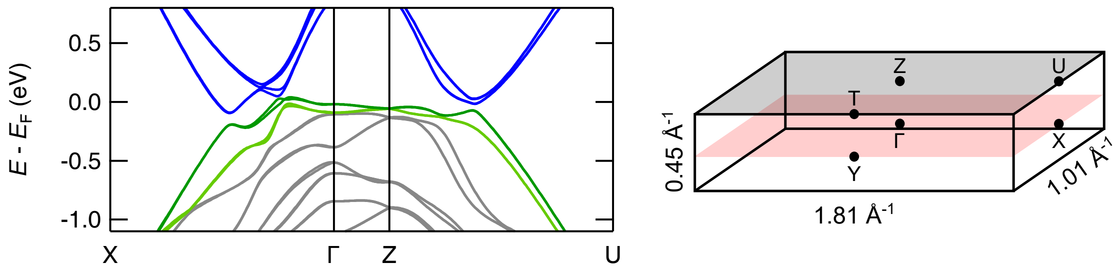

Supplementary Fig. 12. Results of the band structure calculation of Td-WTe_2 for the Γ-X, Γ-Z, and Z-U direction. The 3D Brillouin zone of Td-WTe_2 is shown to the right.

Gaussian with a width of 40 meV (FWHM) (Supplementary Fig. 11b) to simulate the PE signal (Supplementary Fig. 11c). The oscillation amplitude of the peak intensities (IM) was adjusted until the relative amplitude of the simulated PE intensity oscillation yielded a value of $\pm 0.6\,\%$ in agreement with the experimental data from ROI C shown in Fig. 4d of the main text. The simulated PE intensity transient and its corresponding energy-dependent Fourier amplitude and phase signals at the modulation frequency of 0.23 THz are exemplary shown in Supplementary Figs. 11d and 11e for scenario (ii).

SUPPLEMENTARY NOTE 11: BAND DISPERSION ALONG Γ-Z

In the main text, we discuss the potential relevance of the dispersion of the two low energy bulk bands along the Γ-Z direction. For comparison, we show in Supplementary Fig. 12 results of the band structure calculation for the Γ-X, Γ-Z, and Z-U direction. We used the same color coding for the bands as introduced in the main text.

REFERENCES

[1] Sie, E. J. et al. An ultrafast symmetry switch in a Weyl semimetal. *Nature* **565**, 61–66 (2019).

[2] Dai, Y. M. et al. Ultrafast carrier dynamics in the large-magnetoresistance material WTe_2. *Phys. Rev. B* **92**, 161104(R) (2015).

[3] Bauer, M., Marienfeld, A. & Aeschlimann, M. Hot electron lifetimes in metals probed by time-resolved two-photon photoemission. *Prog. Surf. Sci.* **90**, 319–376 (2015).

[4] Kong, W. D. et al. Raman scattering investigation of large positive magnetoresistance material WTe_2. *Appl. Phys. Lett.* **106**, 081906 (2015).

[5] Jiang, Y. C., Gao, J. & Wang, L. Raman fingerprint for semi-metal WTe_2 evolving from bulk to monolayer. *Sci. Rep.* **6**, 19624 (2016).

[6] Song, Q. et al. The In-Plane Anisotropy of WTe_2 Investigated by Angle-Dependent and Polarized Raman Spectroscopy. *Sci. Rep.* **6**, 29254 (2016).

[7] He, B. et al. Coherent optical phonon oscillation and possible electronic softening in WTe_2 crystals. *Sci. Rep.* **6**, 30487 (2016).

[8] Bruno, F. Y. et al. Observation of large topologically trivial Fermi arcs in the candidate type-II Weyl semimetal WTe_2. *Phys. Rev. B* **94**, 121112(R) (2016).

[9] Zeiger, H. J. et al. Theory for displacive excitation of coherent phonons. *Phys. Rev. B* **45**, 768–778 (1992).

[10] Garrett, G. A., Albrecht, T. F., Whitaker, J. F. & Merlin, R. Coherent THz Phonons Driven by Light Pulses and the Sb Problem: What is the Mechanism? *Phys. Rev. Lett.* **77**, 3661–3664 (1996).

[11] S. Hüfner, *Photoelectron Spectroscopy - Principles and Applications*, 3rd ed. (Springer, Berlin Heidelberg, 2003).
